# Detection and Control Framework for Unpiloted Ground Support Equipment within the Aircraft Stand

**DOI:** 10.3390/s24010205

**Published:** 2023-12-29

**Authors:** Tianxiong Zhang, Zhiqiang Zhang, Xinping Zhu

**Affiliations:** Air Traffic Management College, Civil Aviation Flight University of China, Deyang 618307, China; tianxiongzhang@cafuc.edu.cn (T.Z.);

**Keywords:** autonomous driving, Unpiloted Ground Support Equipment, multi-sensor fusion, computer vision

## Abstract

The rapid advancement in Unpiloted Robotic Vehicle technology has significantly influenced ground support operations at airports, marking a critical shift towards future development. This study presents a novel Unpiloted Ground Support Equipment (GSE) detection and control framework, comprising virtual channel delineation, boundary line detection, object detection, and navigation and docking control, to facilitate automated aircraft docking within the aircraft stand. Firstly, we developed a bespoke virtual channel layout for Unpiloted GSE, aligning with operational regulations and accommodating a wide spectrum of aircraft types. This layout employs turning induction markers to define essential navigation points, thereby streamlining GSE movement. Secondly, we integrated cameras and Lidar sensors to enable rapid and precise pose adjustments during docking. The introduction of a boundary line detection system, along with an optimized, lightweight YOLO algorithm, ensures swift and accurate identification of boundaries, obstacles, and docking sites. Finally, we formulated a unique control algorithm for effective obstacle avoidance and docking in varied apron conditions, guaranteeing meticulous management of vehicle pose and speed. Our experimental findings reveal an 89% detection accuracy for the virtual channel boundary line, a 95% accuracy for guiding markers, and an F1-Score of 0.845 for the YOLO object detection algorithm. The GSE achieved an average docking error of less than 3 cm and an angular deviation under 5 degrees, corroborating the efficacy and advanced nature of our proposed approach in Unpiloted GSE detection and aircraft docking.

## 1. Introduction

Globally, airport authorities are increasingly focused on improving safety and efficiency while reducing labor costs through technological innovations. The growing use and sophistication of Unpiloted Robotic Vehicles (URVs) have notably captured the civil aviation industry’s interest in adopting such technologies within the controlled environments of airports [1]. This interest is particularly concentrated on integrating Unpiloted Ground Support Equipment (GSE) to streamline flight operations in airside areas [2]. Traditional GSE operations, reliant on human drivers, are prone to fatigue and human error. These factors contribute to a heightened risk of vehicle-to-aircraft collisions, especially during periods of heavy traffic, poor visibility, or nighttime operations. According to the International Air Transport Association (IATA), incidents involving GSE and aircraft collisions or unintended contacts lead to annual losses of around USD 4 billion, with aircraft doors being the most frequently damaged components [3]. The primary cause of these incidents has been identified as human errors by flight crews and ground service personnel [4]. Moreover, conflicts in apron operations result in global financial losses exceeding USD 10 billion annually [5]. Implementing unpiloted technology is seen as a solution to enhance efficiency and raise safety standards in apron operations.

Airport unpiloted driving equipment represents the latest generation of airport technology, equipped with sophisticated sensors, controllers, and other components. This equipment leverages cutting-edge technologies such as artificial intelligence, enabling complex environmental perception, intelligent decision making, collaborative control, and other autonomous driving functions. These functionalities are crucial for ensuring safe operations within the airport vicinity [6]. As airports increasingly move towards unpiloted operations, advancing Unpiloted GSE in areas like apron aircraft stands and aircraft cabin door docking areas poses significant challenges. These challenges include:

- Potential conflicts between Unpiloted GSE processes and other entities (vehicles or personnel).
- The need for collision avoidance control during Unpiloted GSE operations, considering the interactions of all entities within the apron aircraft stand.
- The high precision and safety requirements for Unpiloted GSE when docking at the cabin door.

This study explores the navigation and docking control of Unpiloted GSE within aircraft stands. Regarding navigation control, the paper introduces a methodology for designing virtual channels specifically for Unpiloted GSE within a single aircraft stand. This design includes turning induction markers to guide control within these channels. Additionally, the study proposes an algorithm for detecting the boundary line of virtual channels in this context. Moreover, it outlines a set of obstacle avoidance algorithms tailored to the unique requirements of Unpiloted GSE within aircraft stands.

Concerning docking control, the research initially presents a vehicle pose control method for the docking process, based on the enhanced YOLO algorithm [7,8,9] and fuzzy PID control [10]. In compliance with civil aviation GSE docking standards, the paper then suggests strategies for docking control during the Unpiloted GSE docking process.

**Figure 1:**
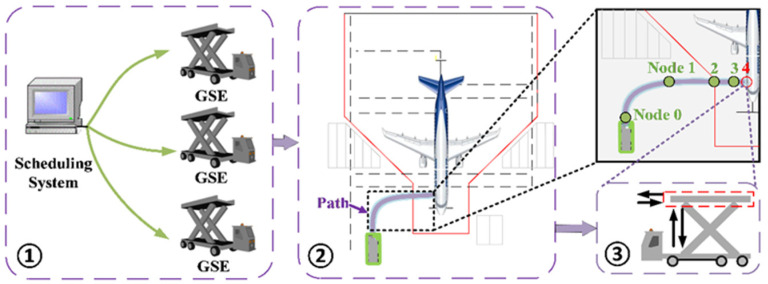
Unpiloted GSE operation workflow.

Assuming a centralized dispatching system that conveys flight service directives to the Unpiloted GSE, these directives include detailed information about the aircraft type (as shown in part ① of Figure 1). This encompasses flight service data and operation start and end times. The process of GSE navigation and docking within the aircraft stand follows the sequence depicted in part ② of Figure 1. Specifically, the vehicle begins navigation from Node 0, performs brake tests at Node 1, slows to a preparatory docking speed at Node 2, further decelerates to docking speed at Node 3, and eventually comes to a stop. The operational procedures of the GSE platform, occurring while the vehicle is stationary (i.e., at Node 4), are not covered in this study.

### 1.1. Contributions

To summarize, the main contributions of the article are as follows:

- A novel methodology is introduced for designing virtual channels within an aircraft stand specifically for Unpiloted GSE. This includes the creation of GSE turning induction markers, aimed at optimizing the navigation of GSE towards specific docking positions tailored for different aircraft models.
- We present a perception algorithm tailored for the autonomous navigation of GSE. This algorithm is capable of accurately detecting the boundaries of virtual channels, identifying obstacles, and recognizing docking doors in real time.
- A comprehensive vehicle control algorithm for GSE operating within an aircraft stand is proposed. This encompasses advanced obstacle avoidance strategies and detailed docking control algorithms, ensuring precise and safe docking of the GSE.

### 1.2. Outline of the Paper

The remaining sections of this paper are organized as follows: Section 2 offers a concise review of the latest research developments in the navigation and docking of GSE. Section 3 delineates the comprehensive framework for detection and control algorithms tailored to the navigation and cabin door docking procedures of Unpiloted GSE. Section 4 presents a methodology for creating virtual channels specifically for Unpiloted GSE. Detailed expositions on the design of detection algorithms for Unpiloted GSE and their control mechanisms are discussed in Section 5 and Section 6, respectively. Section 7 details the experiments conducted on the navigation and cabin door docking of Unpiloted GSE, including an analysis of the results. Finally, Section 8 concludes the paper with a summary of findings and implications.

## 2. Related Work

### 2.1. Progress in Unpiloted GSE Navigation Research

Autonomous driving technology is classified into multiple levels, as defined by SAE International, ranging from Level 1 to Level 5 (L1–L5) [11]. In the context of airport operations, the capabilities of Level 4 (L4) automation are particularly relevant [6]. This level primarily differs from Level 5 (L5) in terms of the operational environment; an airport’s airside, being highly regulated and predictable, contrasts sharply with the complex and uncertain conditions of public roads. This distinction significantly reduces the challenges associated with developing autonomous vehicles. Presently, some airports have implemented Unpiloted GSE, such as autonomous shuttle buses at Singapore Changi International Airport [12], robotic sweeping devices at selected UK airports [13], and automated luggage transport carts at London Heathrow Airport [14].

Despite the emergence of some Unpiloted GSE, the focus of most research has been on aspects like path planning [15,16]. There are limited studies on algorithms for automatic navigation and docking of Unpiloted GSE. Donadio F. et al. [17] discussed tasks necessary for airport robots, including navigation and obstacle avoidance, but did not provide detailed algorithmic solutions. Jovančević I. et al. [18] developed an automatic navigation algorithm for aircraft inspection robots, mainly for identifying aircraft cabin doors and probes, but their study’s navigational scope was restricted to areas around the aircraft. The same problem exists with an unpiloted airport foreign object debris detection vehicle using Lidar technology proposed by Elrayes A et al. [19], which needs to follow a fixed track. Kim C. H. et al. [20] designed a semi-automated Unpiloted GSE for bird detection and expulsion at airports, but this system requires manual definition of waypoints for patrolling. Lee S. et al. [21] proposed a sensor fusion-based aircraft detection framework using camera and Lidar sensors; however, this framework only provides object detection results for aircraft.

Our proposed framework for Unpiloted GSE advances beyond previous research by not being limited to the vicinity of aircraft stands. It adheres to airport operation protocols, thereby eliminating the need for manual path definition for Unpiloted GSE. Additionally, our comprehensive automation framework integrates camera, Lidar, and IMU modules for enhanced aircraft detection and vehicle automation.

### 2.2. Progress in Automatic Docking Technology Research

Current research on autonomous docking within apron operations remains underdeveloped, having been predominantly concentrated on the automation of passenger boarding bridges. Anderberg et al. [22] introduced a pioneering automated docking approach in 2006, which involved recording the travel path of the boarding bridge when docking with various aircraft types. This method, however, lacks universality and calls for further enhancement. Subsequent to this, in 2018, Glatfelter et al. [23] recommended placing sensors on the boarding gate’s forefront, employing the gathered data for the visual analysis to achieve alignment with the aircraft’s cabin door. Such an approach permits fine-tuning of the boarding gate to align precisely with the aircraft entrance. Both strategies necessitate manual supervision and substantial preparatory data, which constrains their general applicability. There have been attempts to use image processing techniques involving cameras for the identification of cabin doors as well [24]. Notably, in 2017, Perez et al. [25] devised a method that differentiates between aircraft windows and doors by extracting shapes and connecting the midpoints to approximate the bridgehead’s rotation angle using image perspective. This innovation effectively adjusts the boarding bridge trajectory to fit the aircraft’s access point, but it encounters limitations with proximity docking and demands significant computational resources.

Despite these advancements, there remains a discernable gap in research integrating cabin door detection with the docking mechanism. Existing detection methods utilized in prior studies are becoming obsolete, with substantial deficits in precision, real-time execution, and reliability. To address these issues, we introduce our Unpiloted GSE detection and control framework, leveraging the latest breakthroughs in boundary line detection [26,27,28,29,30] and target perception [31,32,33,34,35].

Recognizing the advanced stage of research on the operational processes of GSE platforms during vehicle static phases (as denoted by part ③ of Figure 1), our study primarily focuses on the exploration of the autonomous navigation and docking procedures of Unpiloted GSE at the aircraft stand.

## 3. Framework of the Detection and Control Algorithm for the Navigation and Cabin Door Docking Process

**Figure 2:**
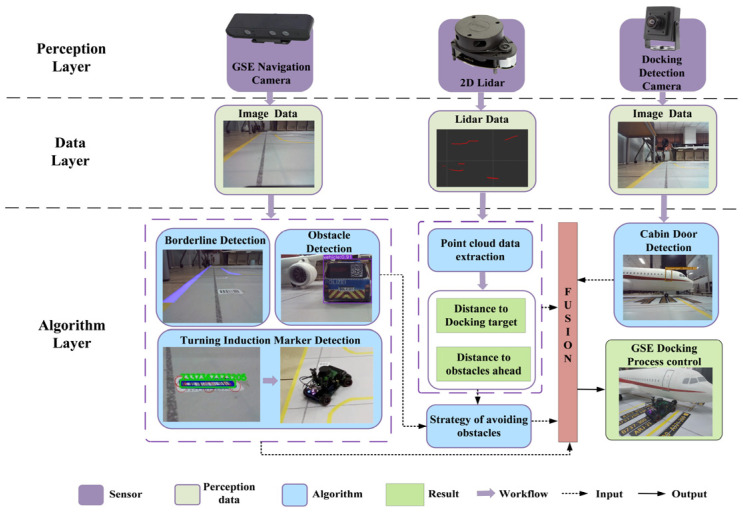
Detection and control algorithm framework for the navigation and docking process of Unpiloted GSE within the aircraft stand.

We have developed an algorithmic framework, utilizing multiple perceptual sensors [36,37], that facilitates the navigation and docking of Unpiloted GSE with the aircraft cabin door within the aircraft stand, as illustrated in Figure 2. It is assumed that prior to executing the algorithm, the Unpiloted GSE has acquired the necessary operational instructions from the airport’s centralized dispatching system.

The control algorithm comprises three essential layers: perception, data processing, and algorithmic processing. At the perception layer, three sensors are employed: the GSE Navigation Camera, 2D Lidar, and the Docking Detection Camera. The data layer processes images captured by the GSE Navigation Camera and the Docking Detection Camera, while the 2D Lidar measures the distance between the GSE and its targets. In the algorithmic layer, a virtual channel boundary line detection algorithm first analyzes images from the GSE Navigation Camera to define the boundaries of the channel and identify turning induction markers, such as magnetic induction road stickers or induction tags (in this study, barcodes were utilized). The same camera also aids in obstacle detection. The Lidar’s point cloud data determine the proximity to the docking target and any obstructions. The Docking Detection Camera primarily detects the docking door itself. This multi-sensor data is integrated and fed into the GSE control algorithm, which guides a PID controller by utilizing virtual channel boundary data to maintain the GSE within a designated path. Furthermore, turning induction markers aligned with the specific aircraft type trigger the PID controller to command the GSE to turn, or proceed to the next marker if applicable. Additionally, in the event of detecting a potential obstacle, the algorithm classifies and calculates its location and size, incorporating distance data from the Lidar to formulate and execute an optimal avoidance maneuver. The detection of the docking door and its distance aids the PID controller in adjusting the GSE’s docking velocity to ensure a successful engagement with the docking process.

## 4. Virtual Channel Layout Method within the Aircraft Stand for Unpiloted GSE

**Figure 3:**
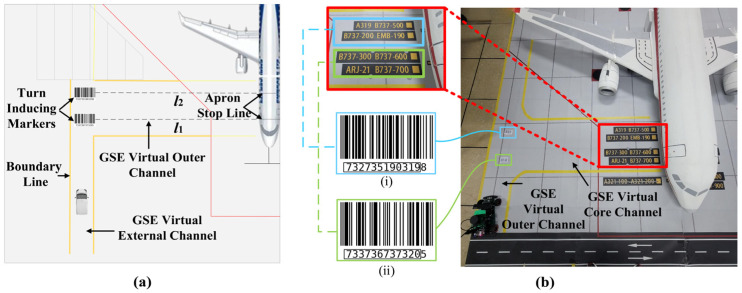
(**a**) Design diagram of the Unpiloted GSE virtual channel; (**b**) virtual channel design scheme implemented in a 1:10 scale apron sandbox of a Class C aircraft stand. Turning induction marker (i) stand for B737-200 B737-500 E190 A319. Turning induction marker (ii) stand for B737-300 B737-600 B737-700 A320.

We have developed a virtual channel within the aircraft stand specifically designed for Unpiloted GSE to enhance its operation and control. As shown in Figure 3, the channel consists of two sections: the GSE virtual outer channel and the GSE virtual core channel. Yellow boundary lines delineate both sections, guiding the GSE along a predetermined path. The design of the GSE virtual outer channel allows for the passage of a single vehicle and incorporates turning induction markers to regulate the GSE’s movements. These markers ensure optimal turning points based on the aircraft type being serviced, minimizing the need for unnecessary vehicle adjustments. The GSE virtual core channel can consist of multiple virtual lanes (e.g., $l_{1}$, $l_{2}$), each related to different stop lines within the aircraft stand. Its width is determined by factors such as the distance between stop lines, the gap between the front passenger door and front cargo door, the required safe distance between vehicles, and the width of the GSE.

When the Unpiloted GSE enters the aircraft stand, GSE first enters the virtual outer channel. Upon reaching a turning induction marker, it verifies whether the identified aircraft type matches the one being serviced. If there is a match, the GSE transitions into one of the GSE virtual core channels. If not, it proceeds to the next turning induction marker. Once inside the virtual core channel, the GSE adjusts its position and orientation based on the location information obtained from the Docking Detection Camera, ensuring alignment with the cabin door.

The implementation of a virtual channel within the aircraft stand effectively organizes the previously chaotic routes of GSE operations [38], reducing the occurrence of accidents stemming from unorganized vehicle movements at the airport. Incorporating turning induction markers allows the Unpiloted GSE to navigate efficiently to the designated door positions of different aircraft, improving operational efficiency. Moreover, this delineation method offers a cost-effective solution for automating GSE operations by simply installing a camera and 2D Lidar on the existing GSE vehicle, eliminating the need for multiple expensive sensors.

## 5. Detection Algorithm for Unpiloted GSE

In this section, we present the detection algorithms utilized, which rely on both image and Lidar data. For video data, we apply an enhanced traditional image processing method to detect the boundary lines of the virtual channel, thus obtaining the location information of the channel boundaries to regulate the vehicle’s navigation within the stipulated virtual channel. An advanced version of the YOLO algorithm is utilized for object detection, including the cabin door, providing vital information about the category, location, and motion of the targets. This information plays a crucial role in enabling the GSE’s autonomous obstacle avoidance and docking pose adjustment. In terms of Lidar data, we extract point cloud distance information to determine the distance from the GSE to the target ahead, which is essential for obstacle avoidance and autonomous docking control.

**Figure 4:**
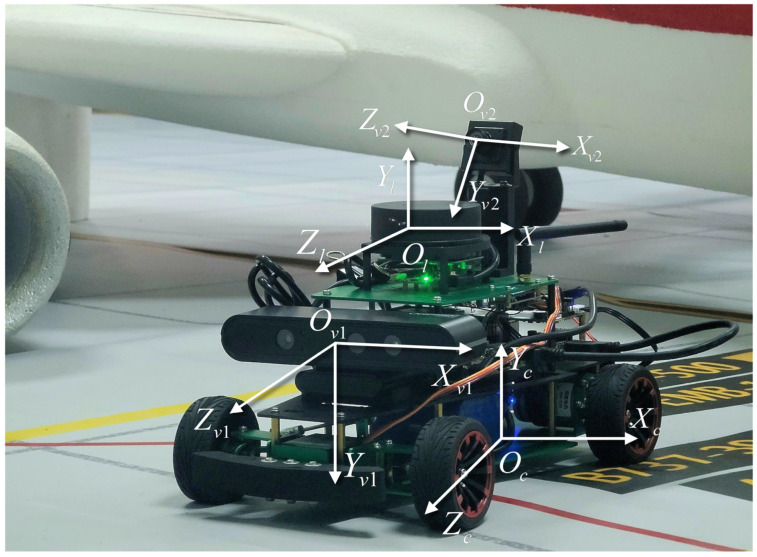
Vehicle detection equipment coordinate system.

Considering that the GSE is equipped with multiple sensors, our initial step involves co-calibration [39] of the camera and the Lidar. Since we utilize 2D Lidar as the ranging sensor, its calibration is accomplished by jointly calibrating the 2D Lidar and camera, based on planar constraints. The coordinate systems are illustrated in Figure 4.

### 5.1. Virtual Channel Boundary Line Detection within the Aircraft Stand

**Figure 5:**
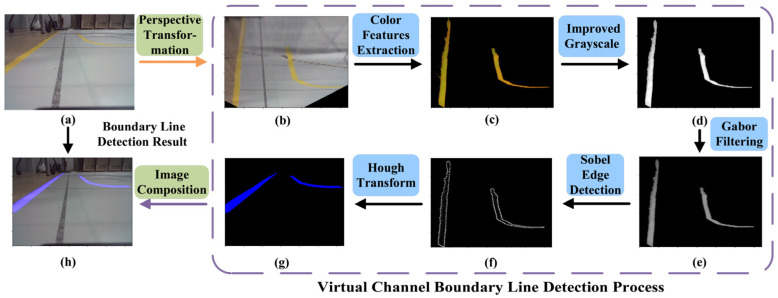
Flowchart of virtual channel boundary line detection algorithm. (**a**) Original image; (**b**) Perspective transformation result; (**c**) Color features extraction result; (**d**) Gray scale processing result; (**e**) Gabor filtering result; (**f**) Sobel edge detection result; (**g**) Hough transformation result; (**h**) Image composition result.

In Section 4, we introduce the concept of the GSE virtual outer channel and the GSE virtual core channel. To enable the GSE’s entry into the designated virtual channel and regulate its navigation within the defined area, yellow boundary lines are implemented around the perimeters of the GSE virtual outer and core channels. This paper presents a boundary line detection method based on traditional image processing techniques, as depicted in Figure 5.

(1) Perspective Transformation: During the GSE’s navigation within the aircraft stand, the boundary lines captured by the camera appear to converge at a specific distance ahead. This convergence poses challenges for the algorithm in detecting the curvature of the virtual channel boundary lines. To address this, we employ a perspective transformation to convert the original image data into a bird’s-eye view, as demonstrated in Figure 5b.
(2) Color Feature Extraction: Our aim is to isolate the target area of the virtual channel boundary lines and minimize the impact of the color of the virtual channel boundary lines and ground features. In the HSV color space, precise segmentation of the specified color can be achieved. The image after color segmentation is presented in Figure 5c.
(3) Enhanced Grayscale: We utilize an advanced grayscale method to enhance the binarized features of the virtual channel boundary lines. This enhancement facilitates edge extraction and Hough transformation, resulting in improved detection accuracy of the virtual channel boundary lines.
(4) Gabor Filtering: Gabor filtering is a texture analysis technique that combines information from both the spatial and frequency domains [40]. By adjusting the kernel size and selecting the appropriate Gabor filter kernel, we can effectively filter the boundary line image information. The filtered image after transformation is illustrated in Figure 5e.
(5) Sobel Edge Detection and Hough Transformation: Prior to edge detection, we perform morphological closing operations to fill minor holes and smooth the object boundaries in the image. Subsequently, an advanced Sobel operator is employed. Considering the directional characteristics of the virtual channel boundary lines in the images captured by the Unpiloted GSE, we select the Sobel operator in the specific direction to extract diagonal edge features. Post-edge detection, an enhanced Hough transformation is applied to extract straight-line information. By incorporating a constraint factor into the traditional Hough transformation, the identified lines are made continuous, ensuring that the angles of the recognized line segments align with the angle features of the virtual channel boundary lines. The extraction effect is illustrated in Figure 5g.

### 5.2. Object Detection in the Aircraft Stand Based on Improved YOLO

**Figure 6:**
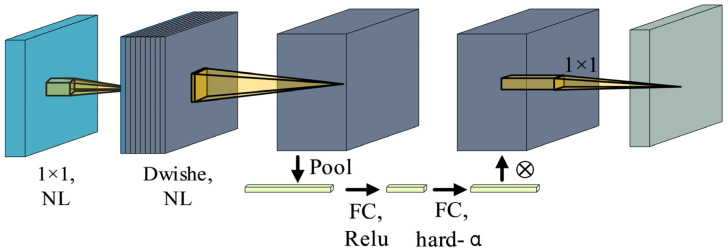
MobileNetV3 block.

The precise docking of Unpiloted GSE to an aircraft door requires rapid pose adjustments, necessitating an efficient real-time object detection algorithm to ensure swift and accurate docking. Similarly, the obstacle avoidance procedure during GSE operations relies on a similar algorithm to enable timely evasion of obstacles and prevent accidents. Therefore, it is essential to implement an object detection algorithm that combines high accuracy with real-time performance. Among comparable single-stage object detection models, YOLOv5 [9] demonstrates commendable detection accuracy, but there is room for improving its real-time operation. In this paper, we propose a lightweight augmentation of YOLOv5 utilizing the MobileNetV3 block [41], aiming to reduce network parameters and enhance the model’s operational speed. The structure of the MobileNetV3 block is illustrated in Figure 6.

To improve the YOLOv5 neck, extraneous layers before average pooling are removed, and 1 × 1 convolution is applied for feature map computation. The adoption of a novel nonlinear h-swish activation function helps retain high-dimensional features while reducing parameters and computational load. In the Backbone component, the Conv module, SPP module, and C3 module are replaced with the MobileNetV3 block, resulting in a significant reduction in the network’s parameter count. Modifications are made to the head structure by removing the original Focus module and introducing the h-swish activation function. This reduces the number of channels in the head, contributing to an overall improvement in network speed. Additionally, an attention mechanism is incorporated into the model to mitigate potential detection accuracy degradation following the network’s optimization.

## 6. Vehicle Control Algorithm Design for Unpiloted GSE within the Aircraft Stand

This section outlines the proposed operating strategy for the GSE. Considering the intricate operating environment of the apron, which influences the control of the GSE in nonlinear and variant manners, it frequently necessitates real-time dynamic adjustments in accordance with operational conditions. Consequently, a fuzzy PID (Proportional, Integral, Derivative) controller [10] is employed to regulate the GSE’s operation.

### 6.1. GSE Navigation Control Algorithm

**(1)**  **Obstacle Avoidance Algorithm Design**

**Figure 7:**
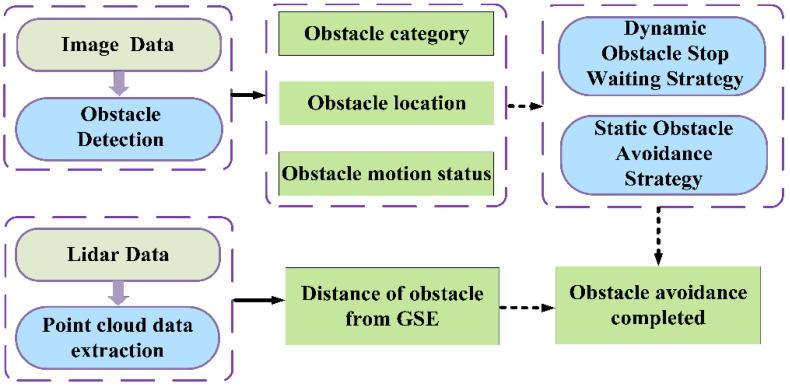
Obstacle avoidance algorithm design schematic.

The operation of Unpiloted GSE requires implementing obstacle avoidance maneuvers to navigate around potential obstructions. Our proposed obstacle avoidance algorithm relies on image data and Lidar point cloud data as inputs. The image data assists in detecting the type, position, and motion state of the obstacle. Within the aircraft stand, typical obstacles include airport personnel and other GSE. For obstacle detection, we employ YOLOv5, and the motion state of the target is determined by tracking its positional changes. In the YOLOv5 algorithm, the center coordinates of obstacles detected in the current and previous frames (denoted as ($x_{1}$,$y_{1}$) and ($x_{0}$,$y_{0}$), respectively) are used to calculate the Euclidean distance *D* between the two positions and the time interval *t* between the frames. From this, the speed of the obstacle can be derived using the velocity formula. Additionally, Lidar point cloud data are primarily utilized to obtain the distance information between the obstacle and the GSE. By assessing the motion state of the obstacle, we can employ either the dynamic or static obstacle avoidance strategy, thereby achieving autonomous obstacle avoidance for the GSE. The algorithm framework is illustrated in Figure 7.

**(2)**  **Dynamic Obstacle Avoidance Strategy**

Assuming the maximum speed of the Unpiloted GSE to be $v_{max}$, and its maximum braking deceleration as $a_{max}$, the minimum distance $D_{min}$, within which the vehicle can halt in the shortest possible time, is derived. For the dynamic obstacle avoidance strategy, the following scheme has been designed: Initially, the obstacle’s target distance for the current and previous frames is obtained via Lidar, which allows for the calculation of the obstacle’s speed relative to the GSE. Subsequently, utilizing the Lidar-measured distance between the obstacle and the GSE, the projected collision time is calculated, assuming no avoidance measures are taken. If the projected collision time is less than the time required for deceleration from the current speed to zero, the Unpiloted GSE should initiate a stop strategy to prevent a collision. The GSE can apply brakes based on the current speed, maintaining a constant maximum braking deceleration until the speed reduces to zero. Once the speed is zero, the GSE should remain stationary until the obstacle departs and surpasses the safety distance of $D_{min}$. If the GSE determines the absence of obstacles on its path, it should resume driving, incrementally increasing its speed to the predetermined path speed.

**(3)**  **Static Obstacle Avoidance Strategy**

The first step involves utilizing Lidar to gather distance information between the vehicle and any upcoming obstacles, while the type of obstacle is determined through an object detection algorithm. Next, we introduce the concept of the Dynamic Field of View [42], which helps identify which obstacles should be considered in the obstacle avoidance strategy. The Dynamic Field of View consists of a sector area with a radius equal to twice the current speed of the vehicle and a fixed angle of 0°. The size of the field of view is recalculated in each frame based on the vehicle’s current speed using the formula

(1) $$R_{view}=2\times v_{current}$$

where $R_{view}$ represents the radius of the field of view, and $v_{current}$ is the current speed of the vehicle.

Subsequently, we integrate the field of view into the obstacle avoidance strategy. Leveraging Lidar and the object detection algorithm, we identify all obstacles within the field of view. For each obstacle, we calculate its offset *p* and actual length *L* relative to the vehicle using the following formulas:

(2) $$p=a\tan\xi_{1}$$

(3) $$L=p+a\tan\xi_{2}$$

where *a* signifies the distance between the vehicle and the obstacle, and $\xi_{1}$ and $\xi_{2}$ represent the sector angles at which the obstacle can be scanned by Lidar.

Next, based on the obstacle’s position and size, along with the width of the vehicle and the safety margin, we compute the necessary steering angle θ and yaw distance *C* for the vehicle to safely avoid the obstacle:

(4) $$\tan\theta =\frac{\left(\frac{W_{veh}}{2cos\theta }+s+p\right)}{a}$$

(5) $$C=\frac{\left(a+\frac{W}{2}\right)}{cos\theta }$$

where $W_{veh}$ is the width of the vehicle, *s* is the safety margin between the vehicle and the obstacle, and *W* is the width of the obstacle. According to the category information of the recognized obstacle obtained by the object detection algorithm (two types of obstacles in the aircraft stand: Vehicle, Person), the obstacle width *W* is set to 4 m or 0.5 m, respectively.

**Figure 8:**
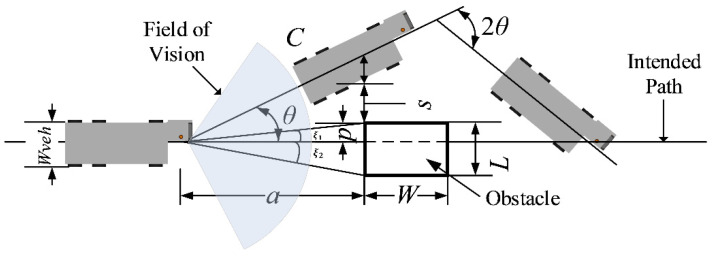
Diagram of static obstacle avoidance strategy (when the obstacle is on the right side of the vehicle).

In each frame, the corresponding avoidance strategy is formulated according to the above computations. If the obstacle is positioned to the right of the vehicle, a leftward turn is executed for avoidance, and vice versa. A detailed illustration of this concept is provided in Figure 8.

### 6.2. Docking Control Algorithm for Unpiloted GSE

**(1)**  **Docking Algorithm Design**

The design of the docking algorithm for the Unpiloted GSE integrates Lidar and an enhanced YOLOv5 model. Initially, Lidar scans the area ahead to determine the distance between the GSE and the cabin door. Meanwhile, the GSE’s navigation speed is determined by a speed sensor. A camera captures the target image of the cabin door, and individual frame images are supplied to the object detection module. This module utilizes the enhanced YOLOv5 model for object detection, generating detection frames as output. The coordinate center of the detection frame is considered the central position of the cabin door. Based on the coordinate information of the cabin door’s center point, the Unpiloted GSE evaluates its position relative to the cabin door (whether it is aligned, to the left, or to the right). This information is then used to fine-tune the vehicle’s direction with the help of a PID controller, ensuring precise docking to the cabin door.

**(2)**  **Docking Control Strategy**

To facilitate the docking of the GSE to the aircraft cabin door, a 2D Lidar is utilized to acquire precise distance measurements. These data allow us to determine the current phase of docking and make the necessary adjustments to the velocity. The docking control strategy consists of a series of sequential nodes, as shown in Figure 1.

- Node 1—15 m stage: When the Unpiloted GSE is 15 m away from the cabin door, we conduct a brake test. This is to ensure that the GSE can stop in a short time in case of emergencies.
- Node 2—4.50 m stage: When the GSE is 4.50 m away from the cabin door, the GSE first decelerates to 5.00 km/h (1.38 m/s). The main purpose of this stage is to ensure that the GSE has enough time for precise docking when it approaches the cabin door.
- Node 3—0.50 m to 0 m stage: When the GSE is 0.50 m away from the cabin door, the GSE performs a second deceleration to 0.80 km/h (0.22 m/s). During this stage, we ensure that the GSE can dock smoothly and precisely to the cabin door through subtle speed and direction control. Upon arrival, the GSE is brought to a complete stop, marking the end of the docking procedure.

Throughout each stage, velocity control directives are transmitted to the PID controller, thereby enhancing both the safety and accuracy of the docking process.

It is worth noting that the control algorithms proposed in this paper are designed in accordance with the Operational Regulations of Ground Support Equipment. The obstacle avoidance algorithms are specifically tailored for common obstacles found at airports, including airport personnel and other GSE. To optimize the efficiency of these obstacle avoidance algorithms, a field of view domain concept is introduced. Additionally, the docking control process incorporates the airport GSE operation rules in the design of an automatic brake test process, an automatic deceleration process, and an automatic angle correction process. These measures aim to achieve a standardized and accurate door docking process.

## 7. Experiment

To minimize interference and mitigate risks associated with on-site validation during airport operations, a 1:10 scale model scenario of a Class C aircraft stand, depicted in Figure 3b, was employed for algorithm validation. The chosen aircraft stand scale model closely mimics the real environment, incorporating accurate proportions and details of the aircraft stand size while accurately representing the connected structural elements. Furthermore, a corresponding aircraft model at a 1:10 scale was utilized to ensure experimental realism. The algorithm is implemented on a Robot Operating System (ROS)-enabled vehicular test platform, which operates on Ubuntu 18.04 and is powered by a quad-core ARM A57 processor clocked at 1.43 GHz with 4 GB of RAM. It is important to note that different hardware configurations may show varying performance. The test vehicle, also at a 1:10 scale, is equipped with a suite of sensors including 2D Lidar equipment, an RGBD camera, a distortion-free RGB camera, an odometer, and an IMU module, as illustrated in Figure 4. The 2D Lidar equipment used is the RPLIDAR A1M8, which has a measurement radius of 0.1–12 m, a sampling frequency of 8 K, and an angular resolution of <1°. Specific device information is provided in Appendix A.

### 7.1. Verification of Detection Algorithm for Unpiloted GSE

**(1)**  **Experiment on Virtual Channel Boundary Line Detection**

Firstly, the boundary line detection algorithm’s parameters were designed with careful consideration. The color processing threshold of HSV was established based on the yellow virtual channel boundary lines allocated within the aircraft stand, as detailed in this study. The specific threshold design is presented in Table 1.

In the Gabor filter, a kernel size of 7 × 7 was experimentally chosen, and six different Gabor filter kernels were generated in the angle interval π at a step of π/6. For the lambda parameter, ten different filter kernels were generated within 2 to 4 at a step of 0.2. The filter kernel with the best detection results was chosen, which had an angle of π/2 and a lambda of 3.

To evaluate the performance of the proposed algorithm, an experimental dataset consisting of 1000 frames was constructed. The image size of this dataset is 640 × 480, recorded at a rate of 24 frames per second.

The detection algorithm demonstrated remarkable effectiveness in most experimental scenarios. Specifically, it accurately detected 89% of the frames, missed less than 5% of the frames, falsely detected 3% of the frames, and failed to detect the remaining 3% of frames. The algorithm can handle challenging scenes, such as those under low light and exposure conditions. The average computation time on the ROS vehicle was less than 100 milliseconds, excluding camera read and decoding time.

**Figure 9:**
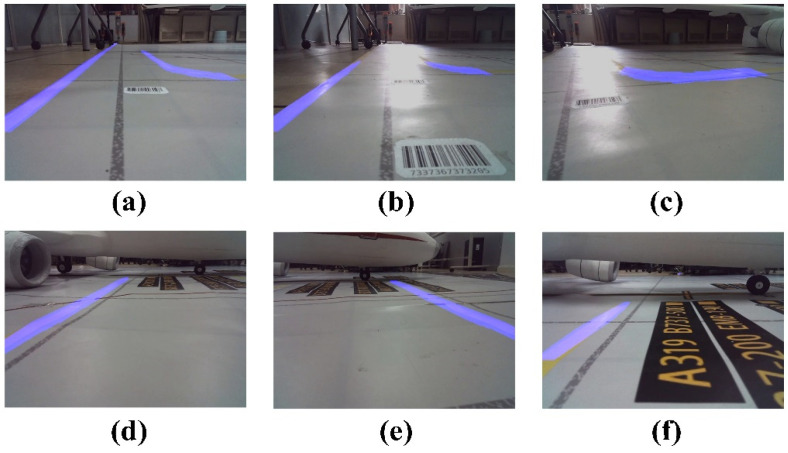
Successful instances of virtual channel boundary line detection. (**a**) Case 1 of successful detection of GSE Virtual Outer Channel boundary lines;(**b**) Successful detection case of GSE Virtual Outer Channel boundary line under exposure conditions; (**c**) Case of successful detection of boundary lines during turning; (**d**) Case 1 of successful detection of GSE Core Channel boundary lines; (**e**) Case 2 of successful detection of GSE Core Channel boundary lines; (**f**) Case 3 of successful detection of GSE Core Channel boundary lines.

**Figure 10:**
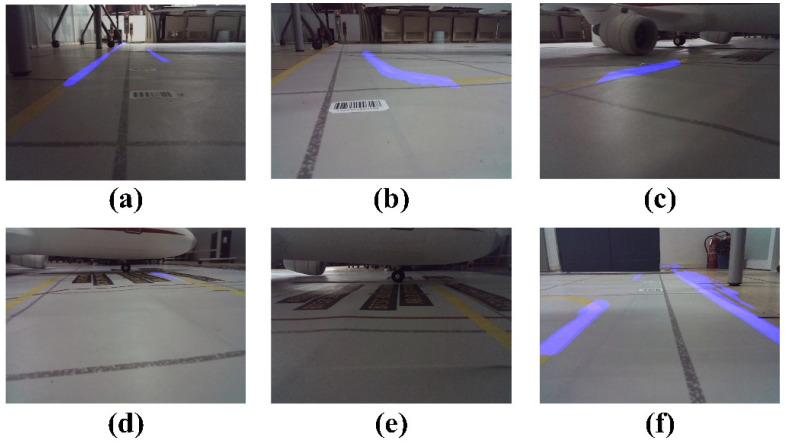
Unsuccessful instances of virtual channel boundary line detection. (**a**) Case 1 of missed detection; (**b**) Case 2 of missed detection; (**c**) Case 3 of missed detection; (**d**) Case 1 of detection failure; (**e**) Case 2 of detection failure; (**f**) Case of false detection.

The algorithm’s computation results are illustrated in Figure 9 and Figure 10. Poor detection results were classified into three categories: missed detection, false detection, and detection failure. According to the literature [43], missed detection refers to instances wherein more than 10% of the virtual channel boundary lines in the image are undetected, false detection refers to instances wherein sections of the image not belonging to the virtual channel boundary lines are detected, and detection failure refers to instances wherein the virtual channel boundary lines are completely undetected. Figure 10a–c represent missed detection, Figure 10d,e represent detection failure, and Figure 10f illustrates false detection.

During the experiment, an unusual instance of false detection occurred, wherein a section of the floor was erroneously identified as the virtual channel’s edge, as illustrated in Figure 10f. However, this did not affect our vehicle’s performance since the inner edge of the channel was fully recognized. Considering that the GSE does not interact with the boundary’s inner edge during operation, the majority of missed detections were primarily attributed to poor lighting conditions in the environment (Figure 10a,c). The suboptimal detection outcomes largely stemmed from the exceptionally low contrast of the markings delineating the virtual channel boundaries. From a human perspective, the virtual channel boundary lines can sometimes be challenging to discern (Figure 10c). However, it is worth noting that the algorithm proposed in this paper effectively handles other challenging lighting conditions (Figure 9b,c). When the vehicle is turning, the absence of an anti-shake algorithm in the camera, combined with high speed, substantial image shift, and significant distance from the virtual channel boundary lines, can potentially result in detection failure (Figure 10d). Therefore, further research is warranted to improve the detection of virtual channel boundary lines under these specific conditions.

The quantitative results are summarized in Table 2.

**(2)**  **Experiment on Turning Induction Marker Detection**

**Figure 11:**
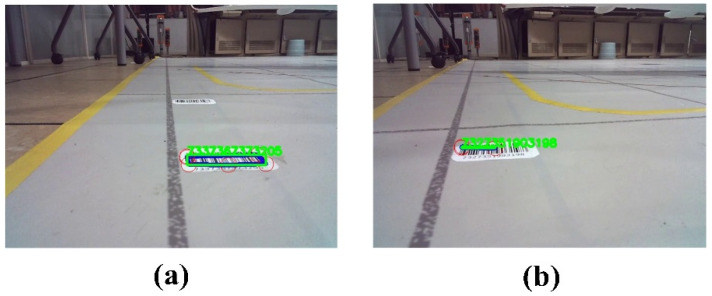
Detection of turning induction markers. (**a**) Case 1 of turning induction marker detection; (**b**) Case 2 of turning induction marker detection.

Figure 11a,b illustrate the detection results of two different turning induction markers. Across 30 experimental trials conducted under diverse environmental conditions, we achieved remarkable detection performance, with a success rate surpassing 95%. Only one instance of detection failure occurred under dimly lit conditions. Considering the practicalities of apron scenarios, embedding the turning induction markers within the pavement and integrating lighting installations could serve as effective strategies to ensure reliable identification performance.

**(3)**  **Experiment on Object Detection in Aircraft Stands Using Improved YOLO**

**Figure 12:**
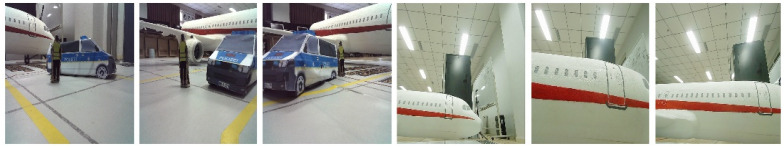
Unpiloted GSE object detection dataset.

To satisfy the operational requirements of Unpiloted GSE, we developed an improved method for the object detection network during GSE operations, as explained in Section 5.2. Acknowledging the scarcity of datasets dedicated to GSE operations within aircraft stands, we simulated the perspective of Unpiloted GSE operations using an aircraft stand sandbox. This simulation enabled the creation of a unique dataset for object detection in aircraft stands by Unpiloted GSE. Comprising 300 images with a resolution of 640 × 480 pixels, this dataset includes three detection object types: Passenger_door, Person, and Vehicle, as illustrated in Figure 12. Considering the relatively small size of this database for neural network-based image processing algorithms, we employed data augmentation to increase the image count, given that the original images already exhibit diverse targets in different poses.

The accuracy of object detection is primarily evaluated based on its *Precision*, *Recall*, and *F*1-Score. In object detection, *Precision* refers to the proportion of correctly detected targets to the total number of detections during Unpiloted GSE operation. *Recall* represents the proportion of samples correctly predicted as positive samples to the actual number of positive samples. Moreover, the *F*1-Score combines *Precision* and *Recall*, serving as a measure to balance these two values. The formula for the *F*1-Score is as follows:

(6) $$F1\text{-}Score=\frac{2Precision\times Recall}{Precision+Recall}$$

**Figure 13:**
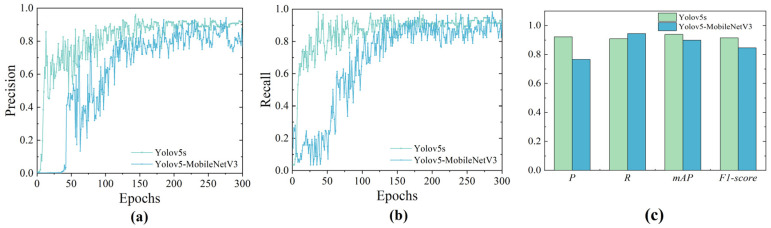
Comparison chart of YOLOv5s and improved YOLO model. (**a**) Precision comparison chart; (**b**) Recall comparison chart; (**c**) Test results comparison chart.

Based on these metrics, experiments were conducted on the YOLOv5s model and the improved YOLO detection model, with the results presented in Figure 13 and Table 3.

The experimental results indicate that the lightweight network employed did not exhibit a significant reduction in *Average Precision* (*AP*) or *F*1-Score. A comparison of the data presented in Table 3 demonstrates a substantial decrease in the computational complexity of the enhanced network, evidenced by a reduction of 14.2 *GFlops*, which represents only 14% of the original value. Regarding the parameters, there is an 80% reduction in the improved network compared to the original YOLOv5s. In summary, the refined YOLOv5 object detection network significantly improves real-time performance without noticeably compromising the model’s detection accuracy and arguably enhances its detection stability. In the context of embedded devices for Unpiloted GSE on the apron, the revised model outperforms the original YOLOv5s model in fulfilling the operational requirements of aircraft stands.

### 7.2. Verification of Vehicle Control Algorithm for Unpiloted GSE

**(1)**  **Verification of GSE Obstacle Avoidance Algorithm**

**Figure 14:**
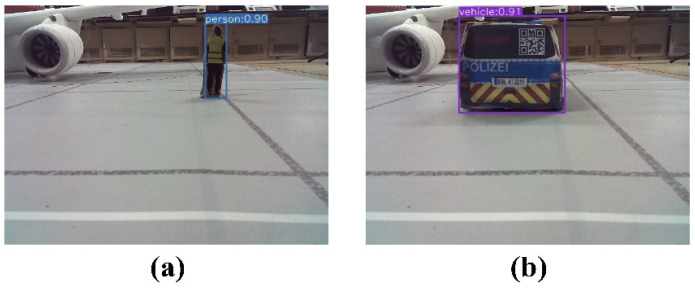
Obstacle detection results. (**a**) Obstacle (Person) detected by the object detection algorithm, (**b**) obstacle (Vehicle) detected by the object detection algorithm.

In this experiment, obstacles within the aircraft stand were categorized into two classes: Person and Vehicle. Figure 14 presents a schematic diagram illustrating the YOLO object detection algorithm’s recognition of obstacles.

Initial experiments concentrated on the dynamic obstacle avoidance strategy, where multiple dynamic obstacles were placed at different distances along the Unpiloted GSE’s path. This setup aimed to simulate various potential obstructions within a real-world airport environment. The results demonstrated that the Unpiloted GSE could successfully execute braking maneuvers upon encountering a dynamic obstacle within its minimum stopping distance ($D_{min}$) and resume navigation once the obstacle moved beyond this safety threshold. Additionally, the experiments revealed that the GSE could still implement effective avoidance actions even when dynamic obstacles suddenly appeared on its path.

**Figure 15:**
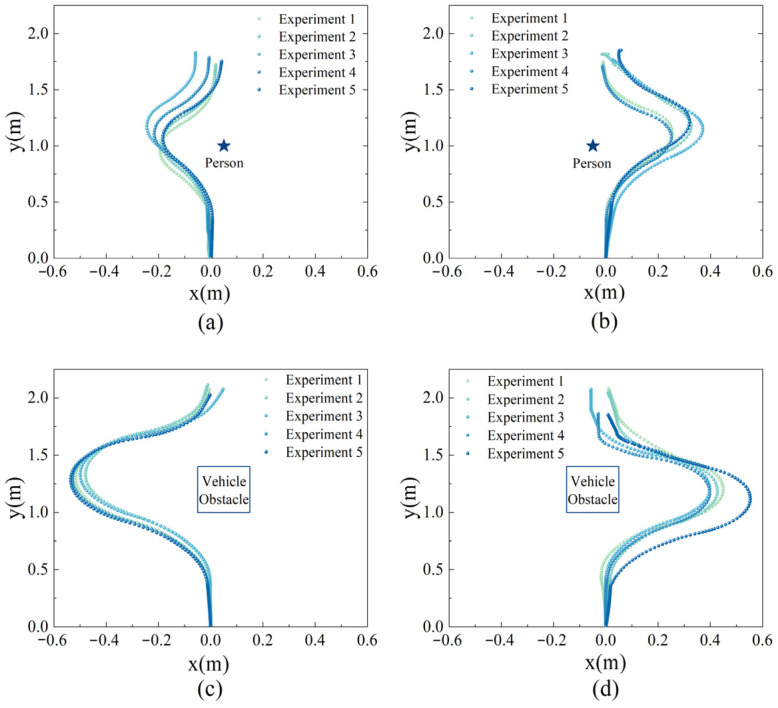
Static obstacle avoidance experiment trajectory chart. (**a**) Obstacle (Person) on the right. (**b**) Obstacle (Person) on the left. (**c**) Obstacle (Vehicle) on the right. (**d**) Obstacle (Vehicle) on the left.

Subsequent experiments assessed the static obstacle avoidance strategy. The analysis of these experiments, depicted in Figure 15, suggests that the strategy can accurately identify obstacle positions and initiate avoidance maneuvers at a predetermined distance (for this experiment, avoidance commenced at a distance of *a* = 0.5 m, corresponding to an actual distance of 5 m, considering that the sandbox’s scale is one-tenth of the actual stand). The vehicle’s turning angle was adjusted based on the obstacle’s length, with a larger angle adopted when navigating around vehicle obstacles to maintain a safe distance, thereby enabling the GSE to re-enter its original virtual channel.

Upon analyzing the experimental results, it was observed that when a vehicle acts as the static obstacle, the outer edge of the obstacle is at a minimum of 0.15 m (corresponding to 1.5 m in real-world conditions), a maximum of 0.26 m, and an average of 0.16 m from the vehicle. Conversely, when a person is the static obstacle, the vehicle’s outer edge maintains a minimum distance of 0.16 m, a maximum of 0.22 m, and an average of 0.17 m from the person. This ensures adherence to the clearance requirement of 1.5 m between the vehicle and the obstacle, as specified by the International Civil Aviation Organization (ICAO) in Annex 14 [44]. Additionally, the experimental data highlighted a certain degree of variation in the vehicle’s turning angle each time. For instance, in Experiment 5 of Figure 15a, the vehicle initiated its turn before reaching the obstacle at a 0.5 m distance, possibly due to the absolute error of 0.02 m in the Lidar. Furthermore, since the experiment was conducted within the confined space of an aircraft stand sandbox, and the vehicle’s turning radius could not be proportionally reduced, this resulted in the vehicle’s turning correction occurring more slowly and the required distance being longer than the actual operational distance.

**(2)**  **Verification of GSE Docking Control Algorithm**

The process of GSE automatic docking with the cabin door relies on real-time positioning within the ROS, using odometer and IMU sensors. The vehicle’s turning angle is calculated and adjusted based on the cabin door’s position until it reaches the docking point. Considering the clearance requirements specified by the ICAO for Ground Support Equipment during aircraft docking [44], and taking into account the experimental environment, scaled proportionally to real airports, the experimental vehicle was considered to have reached the docking position when it was within a 3 cm radius of the docking point.

The location of the vehicle’s Lidar installation, positioned 0.9 m from the vehicle’s leading edge, requires the addition of this distance when assessing the vehicle’s proximity to the cabin door. Moreover, since this research simulates an autonomous lift platform vehicle, the distance between the lift’s front end and the vehicle’s leading edge should be accounted for after the second deceleration. Based on the average GSE size, this distance is standardized to 1 m in this study. Consequently, when the vehicle is 5.4 m from the aircraft, it should decelerate to 5.00 km/h (1.38 m/s); when it is 2.4 m away, it should decelerate to 0.80 km/h (0.22 m/s); and upon reaching a distance of 1.9 m from the aircraft, it should halt. Due to the spatial constraints of the sandbox in the experimental environment, these distances are reduced by a 1:10 scale to align with the sandbox scale. As for vehicle speed, considering the safety of the docking experiment and the braking performance of the experimental vehicle, it is scaled down by a 1:100 factor.

**Figure 16:**
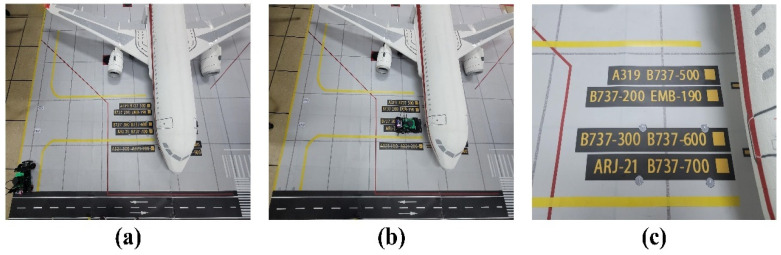
(**a**) GSE initial position; (**b**) GSE docking position; (**c**) target docking point.

Tests were conducted on the automatic docking process. Figure 16a shows the vehicle’s initial position, (b) illustrates the vehicle’s endpoint position after docking with the aircraft, and (c) denotes the standard docking position of the vehicle. The distance error between the actual point and the target point is ±3 cm, which is consistent with the set threshold and meets the industry’s docking accuracy requirements for GSE.

**Figure 17:**
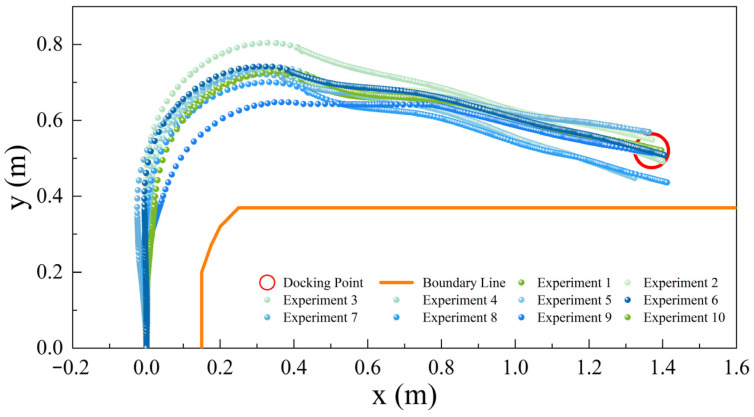
GSE operation trajectory chart.

We plotted the trajectory of the vehicle in ten experiments, as depicted in Figure 17. This figure showcases the vehicle’s trajectory while performing an automatic docking procedure with the cabin door. The X and Y axes in the figure represent the vehicle’s current positional coordinates, considering that the scale of the aircraft stand is one-tenth of an actual aircraft stand. Overall, the vehicle’s movement trajectory is remarkably smooth, ensuring a stable approach to the docking point during actual operations.

However, the consistency of the vehicle’s trajectory in the straight section before turning was not always maintained. This inconsistency arises from the vehicle’s need to adjust its position to remain centrally aligned within the virtual channel. Due to potential errors in the vehicle’s position and orientation each time it is situated at the starting point, the vehicle must rectify its trajectory towards the center of the virtual channel post-initiation.

The vehicle identifies the right-turn position by detecting various turning induction markers. To avoid exceeding the boundary of the virtual channel, the vehicle advances a short distance after recognizing the turning induction marker before executing the right turn. The docking position is typically not directly ahead following the vehicle’s right turn, as indicated by the figure, so the vehicle tends to make a rightward adjustment after the right turn to align with the docking point. Additionally, it was observed that the initial positions of the vehicle’s right turn may not be consistent, possibly due to variations in lighting or other environmental factors during the recognition process, leading to a minor deviation (within 0.1 m) between the actual and ideal paths. However, such a discrepancy is insignificant and can be disregarded.

**Figure 18:**
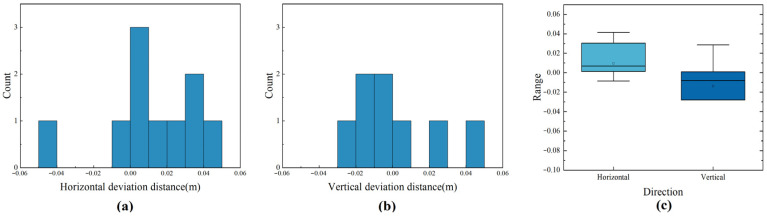
GSE docking position offset analysis chart. (**a**) Docking horizontal deviation statistical chart; (**b**) Docking vertical deviation statistical chart; (**c**) Docking distance deviation box plot.

An error analysis was conducted on the vehicle’s docking point accuracy, as illustrated in Figure 18. Figure 18a,b present histograms of the horizontal and longitudinal errors after docking by the Unpiloted GSE, while Figure 18c shows a box plot of these errors. The experimental data reveal an average horizontal error of 0.02 m, with a maximum of −0.05 m, a minimum of 0 m, and a standard deviation of 0.02. In the longitudinal direction, the average error was 0.03 m, with a maximum of −0.07 m, a minimum of 0 m, and a standard deviation of 0.03. Further examination indicated that errors were more pronounced in the fifth and eighth trials, with the vehicle’s final halt deviating from the docking point by 0.04–0.05 m. This deviation primarily arises from the vehicle’s use of standard 2D Lidar with an inherent measurement error of approximately 0.02 m, and the absence of a GPS module, relying solely on an odometer and IMU for navigation. Such limitations may not fully compensate for human error during the vehicle’s initial placement, potentially resulting in centimeter-level discrepancies in position. Nevertheless, a maximum deviation of 0.05 m falls within operational tolerances and meets the industry’s docking accuracy requirements.

**Figure 19:**
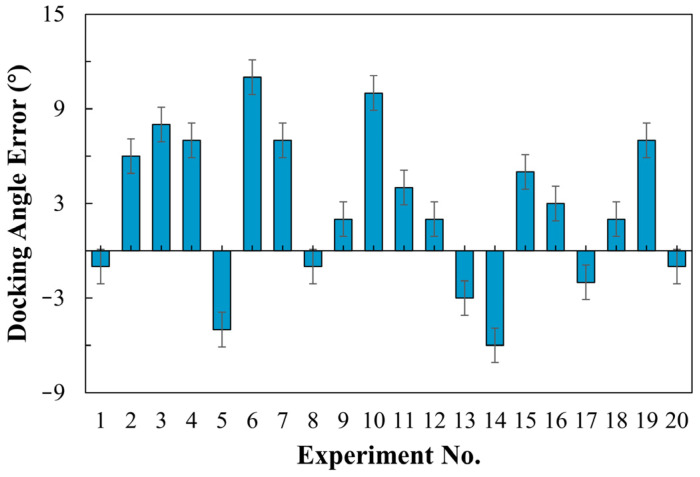
Angle analysis chart after GSE arrives at the docking point.

Figure 19 presents the angular deviation between the vehicle and the aircraft after docking (with positive values indicating right deviation and negative values symbolizing left deviation). Due to the presence of both positive and negative angular fluctuations in this experiment, we conducted twenty trials. The results indicate a deviation ranging from −6° to 11°, with an average of 4.58°. This angular deviation primarily arises from the horizontal displacement between the vehicle and the aircraft door during the terminal docking phase. Despite the vehicle’s continuous angle adjustments, its proximity to the aircraft prevents complete orientation correction, resulting in an angular deviation. The door position data is based on the output of the YOLO detection algorithm, and variations in the image’s center point may lead to residual horizontal errors between the vehicle and the door in the final phase—an issue that requires further investigation. From a practical standpoint, an average deviation of 5° meets the docking precision requirements.

**Figure 20:**
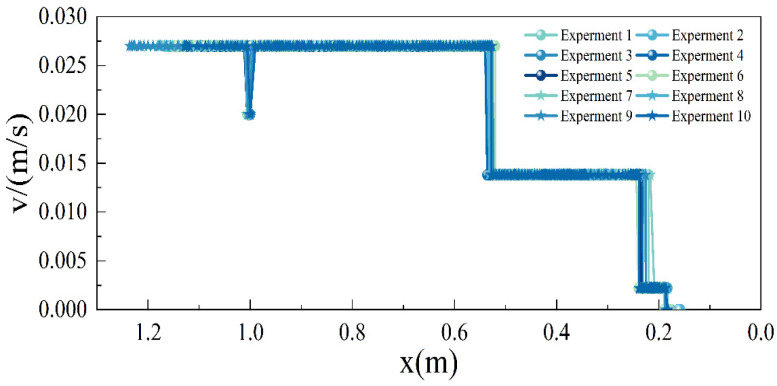
Chart of the relationship between GSE speed and distance to the aircraft.

Figure 20 illustrates the relationship between the vehicle’s speed and distance. Ten experiments were conducted to collect data, and given the minimal fluctuations observed in the experimental results, we consider these ten experiments to be representative of actual operating conditions. As shown in the graph, the vehicle can travel at a speed of 0.027 m/s when it is more than 0.54 m away from the aircraft (corresponding to an actual distance of 5.4 m). The vehicle initiates braking when it is 1 m away from the aircraft (according to actual regulations, the vehicle should take the brake test when it is 15 m away from the aircraft, which corresponds to 1.5 m in the model aircraft stand. However, the vehicle’s turning radius could not be proportionally reduced, resulting in the distance to the aircraft being less than 1.5 m after turning, hence the decision to take the brake test at 1 m). Between the distances of 0.54 m and 0.24 m from the aircraft, the vehicle operates at 0.0138 m/s, decelerating to 0.0022 m/s when the distance is less than 0.24 m, until it halts at a distance of 0.19 m from the aircraft. At the first deceleration, the maximum and minimum distance deviations are 0.02 m and 0.004 m, respectively, with an average deviation of 0.01 m. Similar values are observed at the second deceleration, with the minimum distance deviation being 0 m. These deviations can be primarily attributed to the Lidar’s measurement accuracy of 0.02 m, as all offsets fall within this range. Additionally, the vehicle’s speed remains consistent during operations, indicating effective control over docking speed.

It is important to note that the algorithm verification in this paper was conducted in the aircraft stand sandbox, which may result in some discrepancies compared to real-life scenarios. These differences can be summarized as follows:

1. During the obstacle avoidance experiment, the turning radius of the vehicle cannot be proportionally reduced, resulting in slower correction of the vehicle’s turning and requiring a longer distance than the actual running distance. However, this issue will be alleviated in real-life scenarios.
2. The trajectories of the vehicle in the straight line before turning are not always aligned. This is due to human error in placing the vehicle at the starting position for each experiment, resulting in inconsistencies in the position and angle of the vehicle. Therefore, the vehicle needs to correct its trajectory to the center of the virtual channel after starting. In real situations, this issue will be alleviated.
3. As the vehicle lacks a GPS module, it relies solely on an odometer and IMU for positioning, which introduces some errors. In outdoor scenarios, the vehicle may require the addition of a GPS module to assist in correcting the positioning system, thereby obtaining more accurate navigation and positioning results.

## 8. Conclusions

This study presents a detection and control algorithm for Unpiloted Ground Support Equipment (GSE), enabling automatic navigation to the aircraft and docking with the aircraft cabin door. First, a virtual channel layout method suitable for Unpiloted GSE operation was developed. The introduction of turning induction markers effectively guided the GSE’s automatic progression. Second, to meet the high-precision and real-time pose adjustment requirements for docking with the aircraft cabin door, a detection algorithm incorporating both camera and Lidar data was developed. The application of virtual channel boundary line detection and a lightweight YOLO object detection algorithm facilitated high-precision, rapid detection of virtual channel boundaries, obstacles, and docking door targets. Finally, considering the distinct obstacle characteristics under unique operational scenarios at the aircraft stand and different phases of the docking process, an autonomous obstacle avoidance and automatic docking algorithm were designed for the Unpiloted GSE. Employing a fuzzy PID algorithm enabled precise control of the vehicle’s pose and speed, ensuring safe docking. Experimental validation on a simulated aircraft stand demonstrated excellent coordination among the various modules of our detection and control algorithm, satisfying the preset criteria for docking precision.

In airport environments, Unpiloted GSE may encounter various interference factors. To address common noise and other interferences in airport environments, we have employed a series of advanced techniques at the data processing stage. For the detection of boundary lines, we utilized state-of-the-art image processing techniques, including improved grayscale conversion and the Gabor filter algorithm. These algorithms are specifically designed to handle noise and variations in lighting conditions, ensuring reliable and precise detection results. We have validated these techniques through experiments conducted under challenging conditions such as low light and exposure. For object detection, we employed the YOLO algorithm, which is well-known for its high accuracy and robustness in real-time object detection tasks. We trained the YOLO model using a dataset specifically designed to cover various scenarios and environments within the apron area, enabling fast and accurate detection of obstacles and docking targets in noise-intensive environments. To ensure real-time decision making, we optimized the network structure of YOLO, striking a balance between efficiency and accuracy. We leveraged TensorRT parallel computing technology and hardware acceleration to process data quickly and make timely decisions. Furthermore, we can dynamically adjust parameters based on noise levels and environmental conditions, further enhancing the effectiveness of real-time decision making. In the experimental results, we achieved an accuracy of 89% for virtual channel boundary line detection and 95% for turning induction marker detection accuracy, demonstrating the effectiveness of our algorithms in noise-intensive environments. The *F*1-Score of the YOLO object detection algorithm was 0.845, further confirming its accuracy and robustness.

This framework holds significant potential for application in existing airport systems. Firstly, the algorithms used in the framework have been optimized to reduce computational power requirements, making them suitable for embedded devices. The vehicle perception data is obtained from cost-effective sensors such as cameras and 2D Lidar, resulting in low application costs. Secondly, the framework offers excellent integrability. By integrating perception data with the control board and utilizing the vehicle controller, it can be directly incorporated into common GSE vehicles. Finally, the implementation of virtual channels in this framework effectively addresses the issue of chaotic trajectories in apron operations [38], thereby standardizing the operating routes of GSE vehicles and improving operational efficiency. The techniques in this paper can also be used in research related to Urban Air Mobility (UAM) [45,46] and autonomous driving communication [47].

However, this algorithm may face some challenges in actual airport operations. As our design is targeted towards next-generation smart airports, existing airports may require specific adjustments to the apron markings and lines in order to accommodate the operational rules of the Unpiloted GSE proposed in this paper. In addition, the YOLO object detection algorithm demonstrates some discrepancy in the obtained coordinates of the door center point compared to reality. Due to equipment limitations, our virtual channel boundary line detection method necessitates manually designed parameters. Moreover, our experiments were conducted in a simulated environment and did not account for weather or other complex factors in real operations. In the future, we will install radar on vehicles and try to improve the accuracy of the experimental results in a real environment.

## Figures and Tables

**Table 1:** HSV threshold design.

HSV Parameter	Minimum Value	Title 3
H	23	180
S	104	255
V	44	255

**Table 2:** Missed or false detection counts from 1000 frames.

Type	Results
Missed detection (minor)	4 cases, 51 frames
False detection	1 case, 26 frames
Detection failed	3 cases, 30 frames

**Table 3:** Real-time performance comparison table.

	*GFlops*	Parameters
YOLOv5s	16.5	7,068,936
YOLOv5s-MobileNetV3	2.3	1,382,846

## Data Availability

The data presented in this study are available upon request from the corresponding author.

## Appendix A

**Table A1:** Camera parameter table.

Model	JIERRUIWEITONG DF-100
Sensor specifications	1/4 inch
Pixel size	3 µm × 3 µm
Highest resolution	720P
Frame rate	60
Pixel	1 million
Exposure time	33.33 ms/fps
Interface type	USB2.0
Dynamic range	0.051 lux

**Table A2:** Lidar parameter table.

Model	RPLIDAR A1M8
Measure radius	0.1–12 m
Communication rate	115,200 bps
Sampling frequency	8 K
Scan frequency	5.5–10 Hz
Angular resolution	≤1°
Mechanical dimensions (mm)	96.8 × 70.3 × 55

**Table A3:** Development board specifics.

CPU	128-core Maxwell
CPU	Quad-core ARM A57 @ 1.43 GHz
Memory	4 GB 64-bit LPDDR4 25.6 GB/s
Video Encode	4K @ 30 | 4× 1080p @ 30 | 9× 720p @ 30 (H.264/H.265)
Video Decode	4K @ 60 | 2× 4K @ 30 | 8× 1080p @ 30 | 18× 720p @ 30 (H.264/H.265)
Connectivity	Gigabit Ethernet, M.2 Key E
Display	HDMI and display port
USB	4× USB 3.0, USB 2.0 Micro-B
Others	GPIO, I2C, I2S, SPI, UART
Mechanical	69 mm × 45 mm, 260-pin edge connector
